# Synthesis and characterization of hydroxyl-terminated butadiene-end-capped polyisobutylene and its use as a diol for polyurethane preparation

**DOI:** 10.1039/d0ra00132e

**Published:** 2020-03-05

**Authors:** Kangda Wu, Yibo Wu, Shan Huang, Zhifei Chen, Hao Wang, Yuwei Shang, Shuxin Li

**Affiliations:** Beijing Key Lab of Special Elastomeric Composite Materials, Department of Materials Science and Engineering, Beijing Institute of Petrochemical Technology Beijing 102617 China biptwkd@163.com wangh@bipt.edu.cn shangyuwei@bipt.edu.cn lishuxin@bipt.edu.cn wuyibo@bipt.edu.cn; College of Material Science and Engineering, Beijing University of Chemical Technology Beijing 100029 China h18811044437@163.com chenzhifei1615@163.com; Beijing Key Lab of Special Elastomeric Composite Materials Beijing 102617 China

## Abstract

Hydroxyl-terminated telechelic polyisobutylene (PIB) was prepared through living cationic polymerization. A living PIB chain was formed using the *t*-Bu-*m*-DiCuOMe/TiCl_4_ initiating system and then capped with 1,3-butadiene (BD) to prepare chlorine-terminated telechelic PIB. The chlorine-terminated telechelic PIB was then hydrolysed with tetrabutylammonium hydroxide to form hydroxyl-terminated PIB. Nuclear magnetic resonance spectroscopy confirmed hydrolysis completion. The hydroxyl-terminated PIB was subsequently used as a diol to react with 4,4-methylenebis(phenylisocyanate) (MDI) and produce a PIB-based polyurethane, which showed stronger acid resistance, hydrolysis stability and thermal oxidation stability than a commercial polyurethane.

## Abstract 2

We synthesised hydroxyl-terminated PIB through living cationic polymerisation. Then the hydroxyl-terminated PIB was subsequently used as a diol to produce a PIB-based polyurethane.

## Graphical abstract

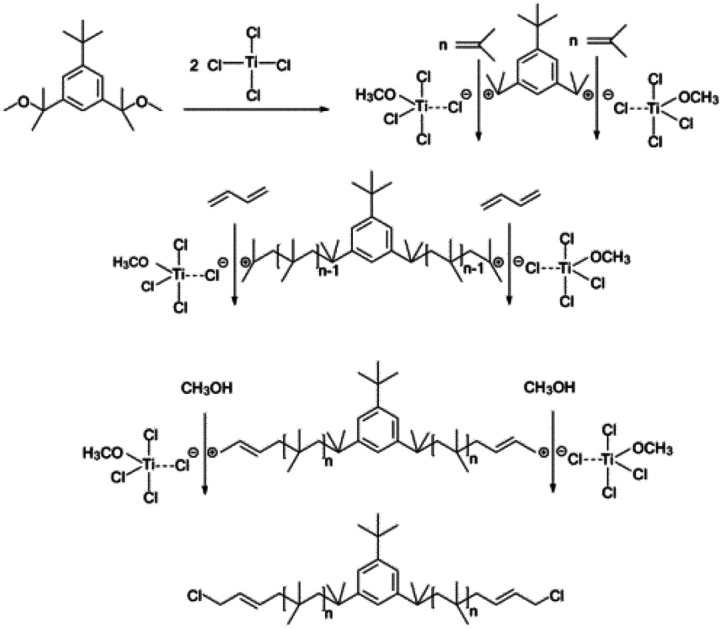

## Introduction

Polyisobutylene (PIB) is a fully saturated hydrocarbon elastomer with superior acid resistance, gas-barrier and mechanical-damping characteristics, oxidative and chemical stability, and biocompatibility.^1–6^ The precise synthesis of hydroxyl telechelic PIB (HO–PIB–OH) compounds has attracted considerable commercial and scientific interest because of their versatility for further chemical modification and synthesis of hydrolysis-resistant polyurethanes.^7–11^ In a recent study, Kennedy *et al.* synthesized HO–PIB–OH from Br–PIB–Br obtained from the quantitative anti-Markovnikov hydrobromination of Allyl–PIB–Allyl.^12,13^ Storey *et al.* utilized thiol-ene click chemistry to produce HO–PIB–OH from telechelic *exo*-olefin-terminated PIB.^14^ Puskas *et al.* utilized direct functionalization with epoxide/TiCl_4_ initiating systems.^15–17^ Wu *et al.* reported the hydrolysis of PIBs end-capped with *tert*-butyl-dimethyl-(4-methyl-pent-4-enyloxy)-silane to obtain HO–PIB–OH.^18^ Faust *et al.* synthesized HO–PIB–OH through the hydrolysis of chloro- and bromoallyl PIBs derived from controlled/living cationic polymerization of isobutylene (IB) and end-capping with BD.^19^ The key to the synthesis of HO–Allyl–PIB–Allyl–OH is the development of a bifunctional initiator that can efficiently initiate the living/controlled polymerization of IB. Therefore, in the present work, we compared the initiation effects of different bifunctional initiators and successfully synthesized HO–Allyl–PIB–Allyl–OH. We also discussed the factors affecting the synthesis of hydroxyl-terminated PIB in detail.

Polyurethane, as an important synthetic material, is characterized by a low relative density, good porosity, high specific strength, and high insulating property. Polyurethane is mainly obtained as a polyester or polyether and, as such, has high moisture permeability due to the disadvantages of the polarity of ester or ether bonds. Therefore, replacement of the soft segment of polyurethane with polymers, such as PIB and poly(tetramethylene oxide) (PTMO), has been attempted to enable the material's application to special environments.^20–29^ Thermoplastic polyurethane ureas (TPUUs) based on PTMO and PIB segments obtained by chain extension of PIB-diamine/PTMO-diol have excellent mechanical properties compared with conventional TPUs. If TPUU-based PTMO and PIB are obtained by incorporating 10–30% PTMO diol into the soft segment, the resulting ultimate tensile strength and elongation at break may be higher than that of conventional TPUs.^30–34^

HO–Allyl–PIB–Allyl–OH has been used as a precursor to synthesize PIB-based polyurethane. In this case, a hydroxyl-terminated telechelic PIB replaces a polyester (ether) polyol that is easily hydrolyzed and pyrolyzed so that the resistance of the resultant material to hydrolysis and heat is greatly improved. The polyurethane molecular chain produced by the reaction of hydroxyl-terminated telechelic PIB and isocyanate contains PIB segments, exhibits the excellent hydrolytic stability of PIB, and has low air permeability and great competitiveness in the market.

## Experimental

### Materials

Titanium tetrachloride (TiCl_4_) (99%; Tokyo Chemical Industry Co.) were dried over phosphorus pentoxide (P_2_O_5_) under N_2_ atmosphere for 12 hours before use, *n*-hexane (99.5%; Beijing Yanshan Petroleum Chemical Co.) were refluxed and distilled with sodium under N_2_ atmosphere for 48 hours before use, 1,4-butadiene (BD) (99%; Beijing Yanshan Petroleum Chemical Co.) and isobutylene (IB) (99.9%; Beijing Yanshan Petroleum Chemical Co.) were condensed in a cold bath in a glove box prior to polymerization. 5-*tert*-Butyl-bis(2-methoxy-2-propyl)benzene (*t*-Bu-*m*-DiCuOMe) was prepared by 5-*tert*-butylisophthalic acid through grignard reaction. Methyl chloride (MeCl) (99%; Beijing Chemical Reagent Co.), commercial polyurethane (PU) (Pellethane5863, Lubrizol, Mn = 10 000 g mol^−1^), 4,4′-methylenebis(phenyl isocyanate) (MDI) (97%; Tokyo Chemical Industry Co.), tetrabutylammonium hydroxide (TBAH) (27wt% solution in H_2_O; Tokyo Chemical Industry Co.), stannous octoate ((Sn(Oct)_2_) (95%; Tokyo Chemical Industry Co.), 1,4-butanediol (BDO) (99%; Tokyo Chemical Industry Co.), tetrahydrofuran (THF) (99.9%; Tokyo Chemical Industry Co.), 2,6-di-*tert*-butylpyridine (D*t*BP) (96%; Tokyo Chemical Industry Co.), methanol (99%; Beijing Chemical Reagent Co.), nitric acid (68%; Beijing Chemical Reagent Co.) were used as received.

### Synthesis of Cl–Allyl–PIB–Allyl–Cl *via* carbocationic polymerization

All polymerizations were carried out under a dry N_2_ atmosphere in a stainless steel glove box using Hex/MeCl (40/60 v/v) solvent mixtures. IB was initially polymerized in the *t*-Bu-*m*-DiCuOMe/TiCl_4_/D*t*BP/−80 °C initiating system for 40 min and then added with the BD stock solution *via* end-capping reaction. After 2 h of reaction, the polymerizations were terminated with prechilled methanol. After solvent evaporation, the product was dissolved in cyclohexane and washed with methanol to remove the uninitiated co-initiator. The processed product was dried at 45 °C in a vacuum oven for 24 h to a constant weight. The yield was 66.45 g (98.7% conversion).

### Synthesis of HO–Allyl–PIB–Allyl–OH

Cl–Allyl–PIB–Allyl–Cl (5.2 g) was dissolved in anhydrous THF (50 mL) at room temperature in a 100 mL round-bottomed flask with vigorous stirring. Then, 12 mL of 27% TBAH (w/w) aqueous solution (0.2 mol) was added to the reaction system. The solution was refluxed overnight under stirring. After 22 h, the polymerizations were terminated with prechilled methanol. After solvent evaporation, the product was dissolved in cyclohexane and washed with methanol to remove the uninitiated TBAH. The processed product was dried at 45 °C in a vacuum oven for 24 h to a constant weight. The reaction yield was 4.91 g (98.2% conversion).

### Synthesis of PIB-based polyurethane

A representative synthesis process of PIB-based polyurethane is described as follows. A mixture of 5.2 g of HO–Allyl–PIB–Allyl–OH (2.60 mmol), 0.44 g of BDO (4.92 mmol), and 1.76 g of MDI (7.02 mmol) was dissolved in 20 mL of anhydrous THF. Then, 5.2 × 10^−3^ g Sn(Oct)_2_ (8.2 × 10^−3^ mmol) was added to the reaction at 65 °C for 4 h. After completion of the reaction, the PIB-based polyurethane was rendered insoluble in organic solvents. The mixture was poured into a 7 × 7 cm^2^ Teflon mold, the residual solvent was evaporated at room temperature, and the product was dried at 50 °C in a vacuum oven for 12 h. Thus, the final product was obtained.

### Measurements

^1^H NMR spectroscopy was applied to understand the structural properties of the monomer and block copolymer composition with a Bruker 500 MHz spectrometer using 5 mm O.D. tubes with the sample concentrations of 5–10% (W/V) in CDCl_3_ or d^8^-THF.

The gel permeation chromatography (GPC) spectra of the polymer for measuring the molecular weights and MWD (*M*_w_/*M*_n_) were obtained using a model 510 HPLC pump, a model 410 differential refractometer, a model 441 UV/visible detector, an online multiangle light-scattering detector and four GPC columns, which were connected in the following series: 500, 10^3^, 10^4^ and 10^5^ Å. THF was used as the eluent at a flow rate of 1.0 mL min^−1^ at room temperature.

FT-IR spectra were obtained using a Shimadzu FTIR 8300 spectrophotometer equipped with a Smart Diamond ATR head at 2 cm^−1^ resolution in the 400 to 4000 cm^−1^ range.

Stress–strain tests were carried on an electronic universal testing machine (Instron3366, USA). The stretch rate was 40 mm min^−1^. The samples (2.0 mm width at the neck, 25 mm long) were shaped with an ASTM standard die. All the samples were tested under room temperature with a load cell of 0.1 kN and a constant cross head speed of 250 mm min^−1^. The final report was the average of three measurements.

## Results and discussion

### Synthesis of Cl–Allyl–PIB–Allyl–Cl *via* living cationic polymerization

**Fig. 1:**
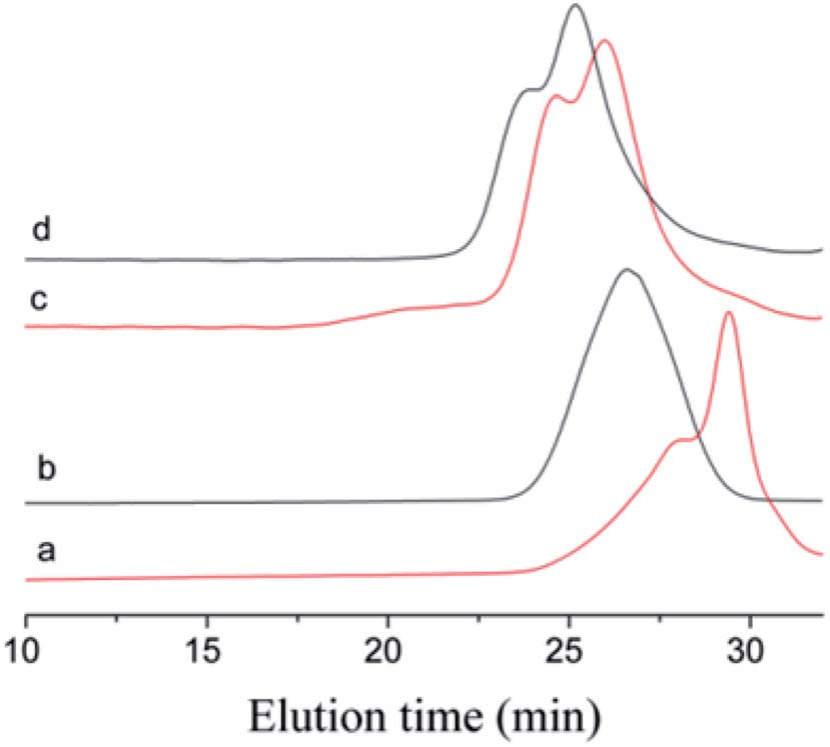
GPC spectra of Cl–PIB–Cl. (a) Initiated by *t*-Bu-*m*-DiCuOMe in the presence of D*t*BP, (b) initiated by *t*-Bu-*m*-DiCuOMe in the absence of D*t*BP, (c) initiated by 1,3-bis-(1-chloro-1-methyl-ethyl)-benzene in the presence of D*t*BP, (d) initiated by 1,3-bis-(1-chloro-1-methyl-ethyl)-benzene in the absence of D*t*BP.

**Fig. 2:**
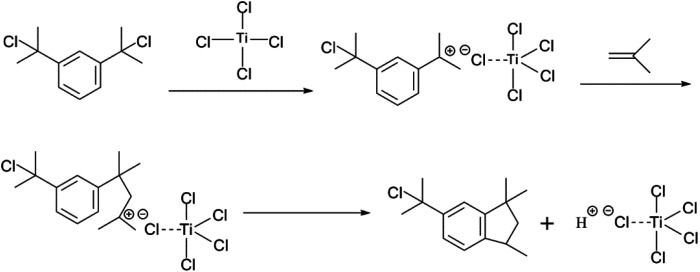
Mechanism of the formation of an indanyl ring of 1,3-bis-(1-chloro-1-methyl-ethyl)-benzene.

**Fig. 3:**
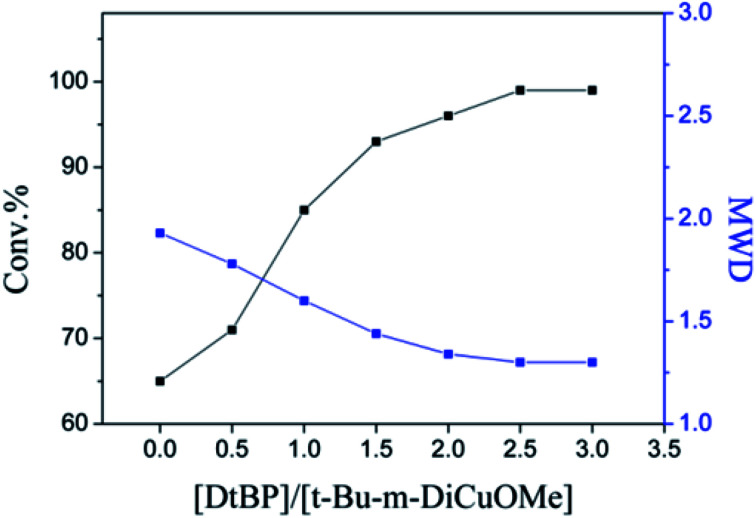
Influence of the ratio of [D*t*BP]/[*t*-Bu-*m*-DiCuOMe] on the polymerization of Cl–PIB–Cl.

The choice of bifunctional initiator is particularly important in the preparation of PIB telechelic polymers. 1,3-Bis-(1-chloro-1-methyl-ethyl)-benzene is a bifunctional initiator used worldwide in the field of carbocationic polymerization. Fig. 1 shows the GPC spectra of PIBs initiated by 1,3-bis-(1-chloro-1-methyl-ethyl)-benzene and *t*-Bu-*m*-DiCuOMe. The GPC spectrum of PIB initiated by 1,3-bis-(1-chloro-1-methyl-ethyl)-benzene in the (c) presence and (d) absence of D*t*BP shows a bimodal peak and wide MWD that may be attributed to the absence of *tert*-butyl in the benzene ring. As shown in Fig. 2, without this bulky group, intramolecular aromatic alkylation occurs with the formation of an indanyl ring, which leads to unacceptable living polymerization.^35^Fig. 1(a) presents the GPC spectrum of PIB initiated by *t*-Bu-*m*-DiCuOMe in the absence of D*t*BP. The GPC spectrum of the polymer shows a bimodal peak, and the molecular weight obtained is remarkably lower than the theoretical value. This finding indicates that chain transfer phenomena occur in the absence of D*t*BP during polymerization. Such phenomena may be attributed to protons from the trace water and β-H at the end of growth chains in the Hex/MeCl reaction system, which can complex with TiCl_4_ to form a proton living center and initiate the uncontrolled polymerization of the IB monomer. As shown in Fig. 1(b), the GPC spectrum of PIB initiated by *t*-Bu-*m*-DiCuOMe in the presence of D*t*BP shows the theoretical molecular weight (*M*_n_ = 9600, MWD = 1.18), which indicates that addition of D*t*BP can maintain carbocation stabilization; intercept protons produced by impurities, water-producing protons, and chain-end β-H growth during the initiation phase; ensure the singularity of living species; and achieve controlled initiation and growth. Thus, D*t*BP can inhibit undesirable side reactions, such as uncontrolled initiation of protons and chain-transition reactions of growing chains with monomers, and maintain living species to continue the efficient initiation of controlled IB polymerization. Fig. 3 illustrates the influence of the ratio of [D*t*BP]/[*t*-Bu-*m*-DiCuOMe] on the polymerization of PIB. Here, the conversion rate increased with increasing D*t*BP content. When the ratio of [D*t*BP]/[*t*-Bu-*m*-DiCuOMe] was 2.5, the conversion rate and MWD reached 100% and 1.3, respectively. In summary, PIB synthesis initiated by *t*-Bu-*m*-DiCuOMe in the presence of D*t*BP can meet our requirements of controllable *M*_n_ and low MWD with high efficiency.

**Fig. 4:**
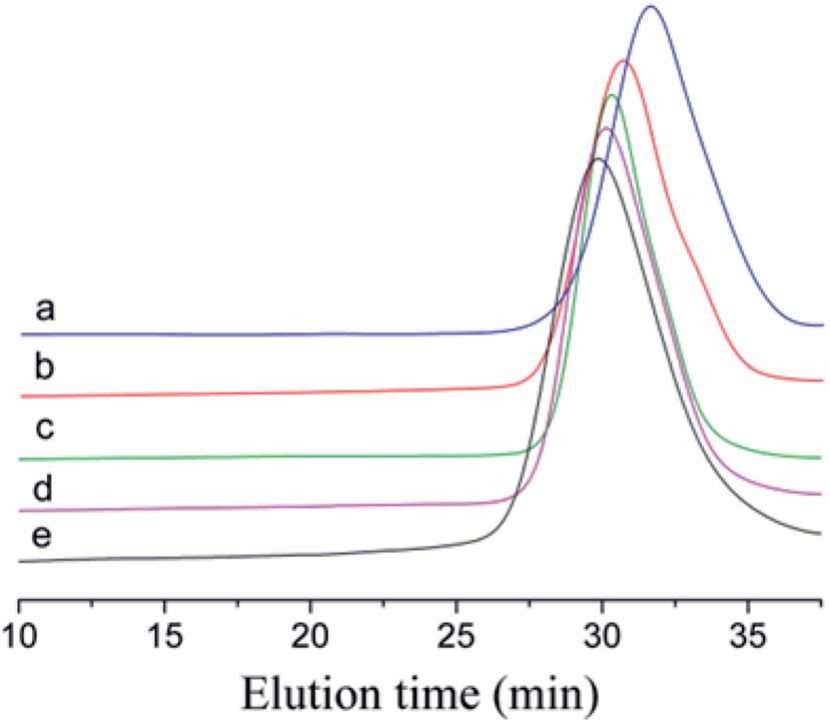
GPC spectrum of Cl–PIB–Cl terminated at (a) 5 min, (b) 10 min, (c) 20 min, (d) 30 min, and (e) 40 min.

**Fig. 5:**
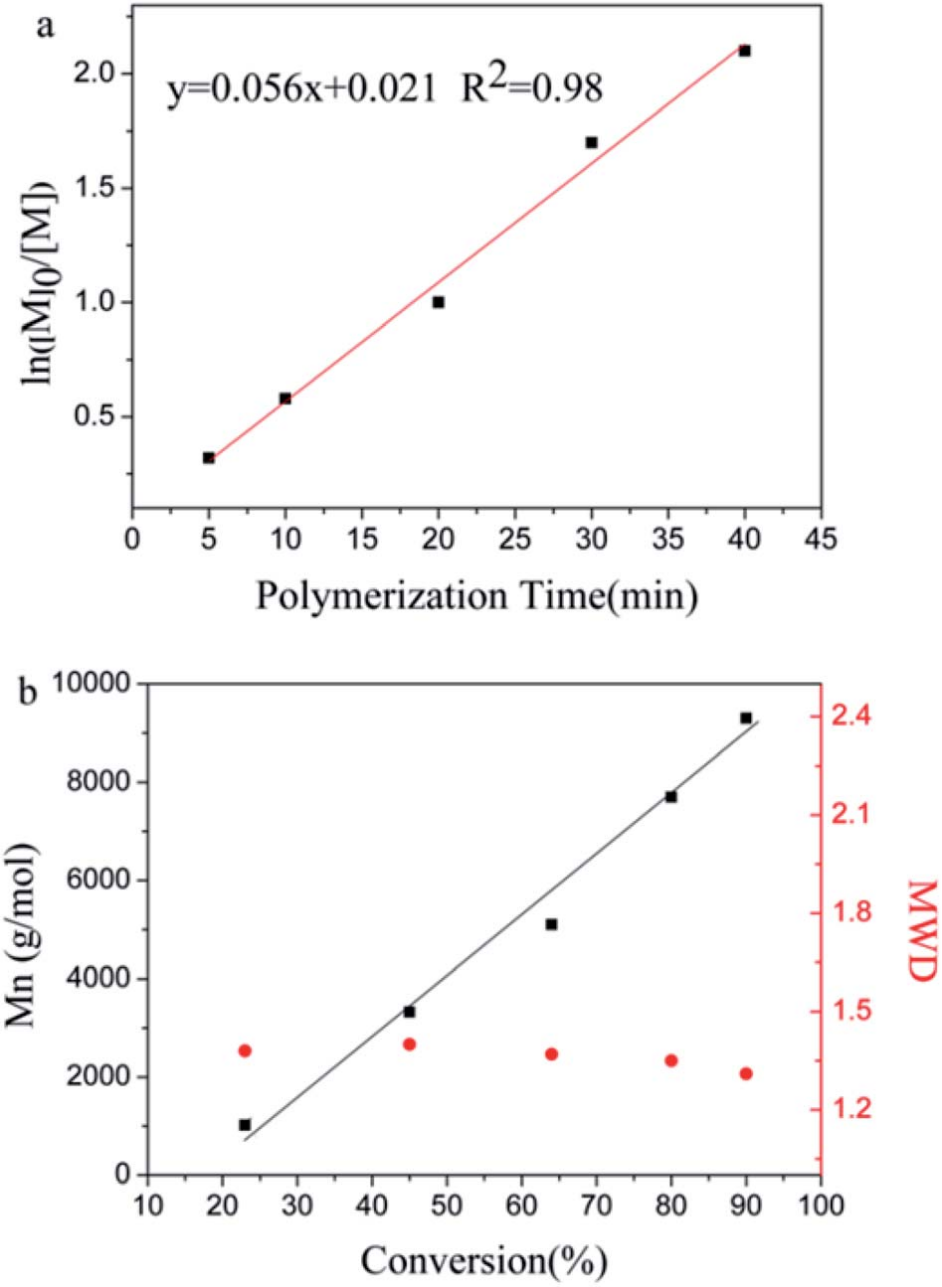
(a) Time-conversion and (b) first-order kinetic plots of the living cationic polymerization of IB.

To prove that the initiating system coupled with *t*-Bu-*m*-DiCuOMe/TiCl_4_ can initiate the living cationic polymerization of IB, we designed a series of polymerization reactions using the same conditions but terminated at different times of 5, 10, 20, 30 and 40 min to analyze their first-order kinetic plots. Fig. 4 shows the GPC spectra of PIBs initiated by *t*-Bu-*m*-DiCuOMe and collected at various times. The PIB peak shifted toward higher molecular weights with increasing polymerization time. All of the polymers are clearly symmetrical and unimodal with a narrow distribution of molecular weights. Fig. 5(a) shows the time-conversion and first-order kinetic plots of the living cationic polymerization of IB. Here, ln([*M*]_0_/[*M*]) varied linearly with the reaction time, thus confirming that the concentration of living centers remains constant.^36^Fig. 5(b) reveals that the Mn of PIB increased in direct proportion to monomer conversion and approached the theoretical value. Moreover, MWD remained constant at nearly 1.2. This behavior validates the supposition that the controlled living cationic polymerization of IB can be synthesized by *t*-Bu-*m*-DiCuOMe/TiCl_4_ in Hex/MeCl and further confirms the reliability of the initiator.

**Fig. 6:**
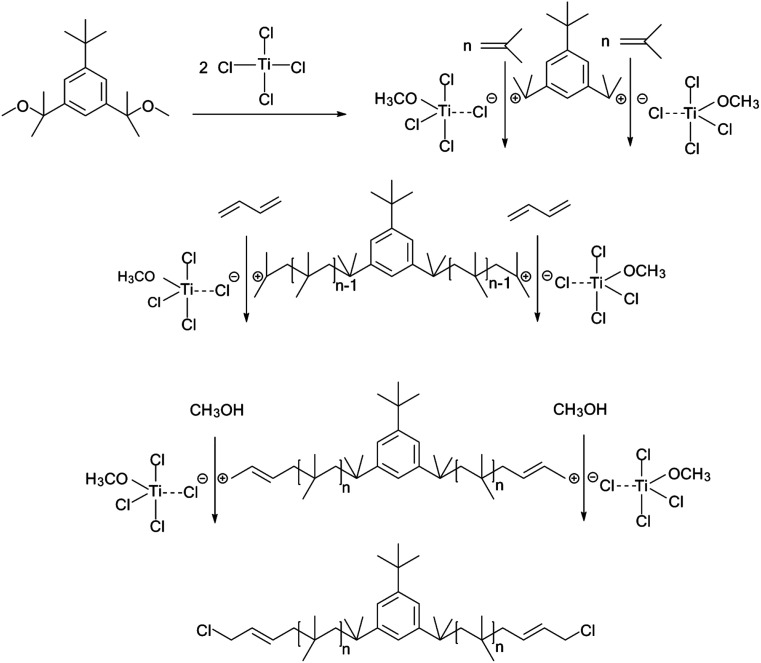
Mechanism of Cl–Allyl–PIB–Allyl–Cl synthesis in the *t*-Bu-*m*-DiCuOMe/TiCl_4_ initiating system *via* carbocationic polymerization in Hex/MeCl 60/40 (v/v) at −80 °C.

**Fig. 7:**
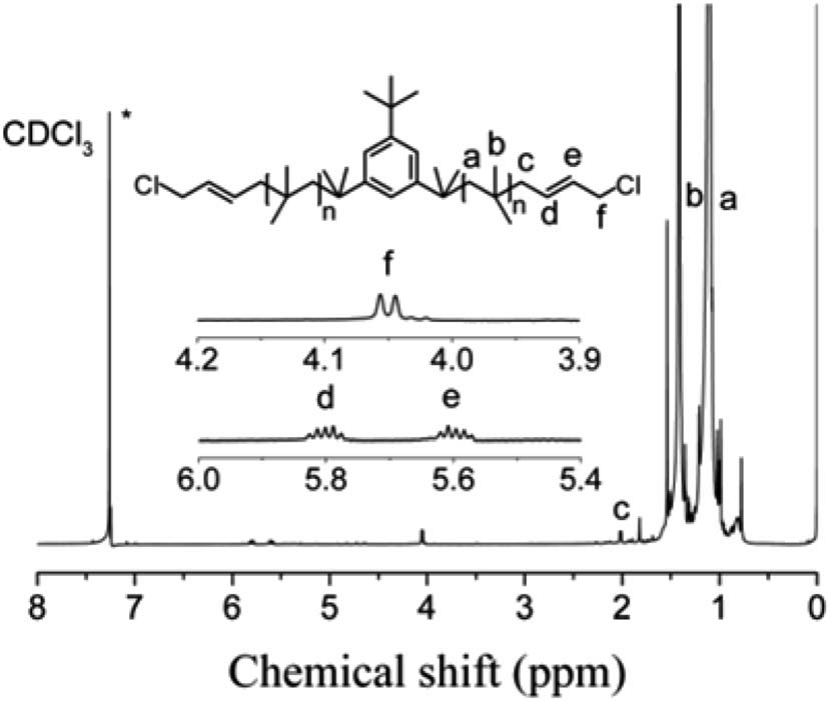
^1^H NMR spectrum of Cl–Allyl–PIB–Allyl–Cl obtained after the capping reaction with BD in Hex/MeCl 60/40 (v/v) at −80 °C. Peaks marked * belong to CDCl_3_. Experimental conditions: *t*-Bu-*m*-DiCuOMe = 7.48 × 10^−3^ mol, TiCl_4_ = 1.34 × 10^−1^ mol, D*t*BP = 1.87 × 10^−2^ mol, IB = 1.2 mol, BD = 7.48 × 10^−2^ mol.

The cationic polymerizations of IB with *t*-Bu-*m*-DiCuOMe/TiCl_4_ initiating systems were conducted in Hex/MeCl 60/40 (v/v) at −80 °C in the presence of D*t*BP. Fig. 6 illustrates the mechanism of PIB. During polymerization, *t*-Bu-*m*-DiCuOMe and TiCl_4_ combine and age to form a living carbocation center. Then, the IB monomer is added and initiated by the living center to form the living end of IB and initiate its chain growth. After 40 min of reaction, BD was added *via* end-capping reaction to attach PIB to both ends of the allylic structure. Finally, methanol is added to quench PIB and form an end-group allyl chlorine. Fig. 7 shows the ^1^H NMR spectrum of Cl–Allyl–PIB–Allyl–Cl obtained *via* living cationic polymerization with the t-Bu-*m*-DiCuOMe/TiCl_4_ initiating system in the presence of D*t*BP and end-capping reaction with BD. A dual characteristic resonance signal at f (4.05 ppm), which corresponds to the H proton in –CH_2_– linked to the PIB group of the terminal allyl chlorine, could be observed. Multiple characteristic resonance signals are shown in d (5.81 ppm) and e (5.61 ppm), corresponding to the H proton in C=C in the PIB terminal BD, and the ratio of characteristic peak areas is c : d : e = 2 : 1 : 1. This result confirms that the end-capping reaction was completed. Thus, on the basis of the ^1^H NMR results, Cl–Allyl–PIB–Allyl–Cl was successfully prepared through cationic polymerization.

### Synthesis of HO–Allyl–PIB–Allyl–OH

**Fig. 8:**
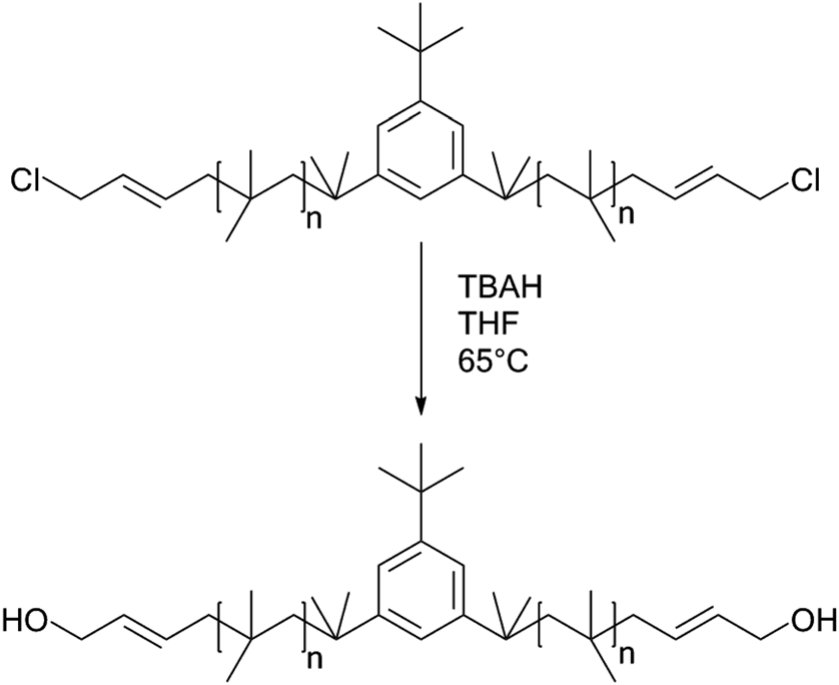
Synthesis of HO–Allyl–PIB–Allyl–OH *via* hydrolysis with Cl–Allyl–PIB–Allyl–Cl.

**Fig. 9:**
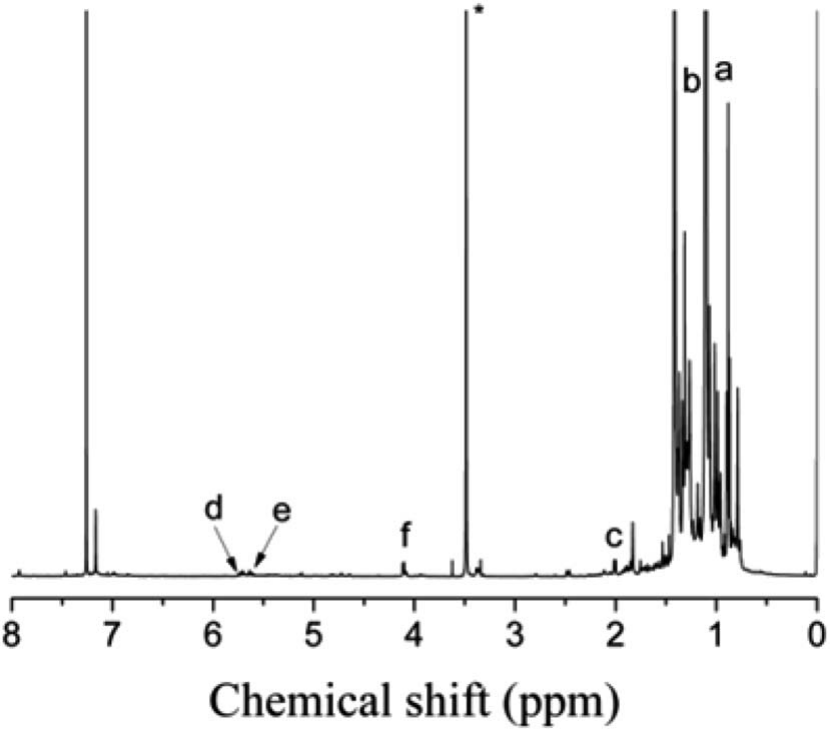
^1^H NMR spectrum of HO–Allyl–PIB–Allyl–OH obtained after hydrolysis. Peaks marked * belong to CH_3_OH.

**Fig. 10:**
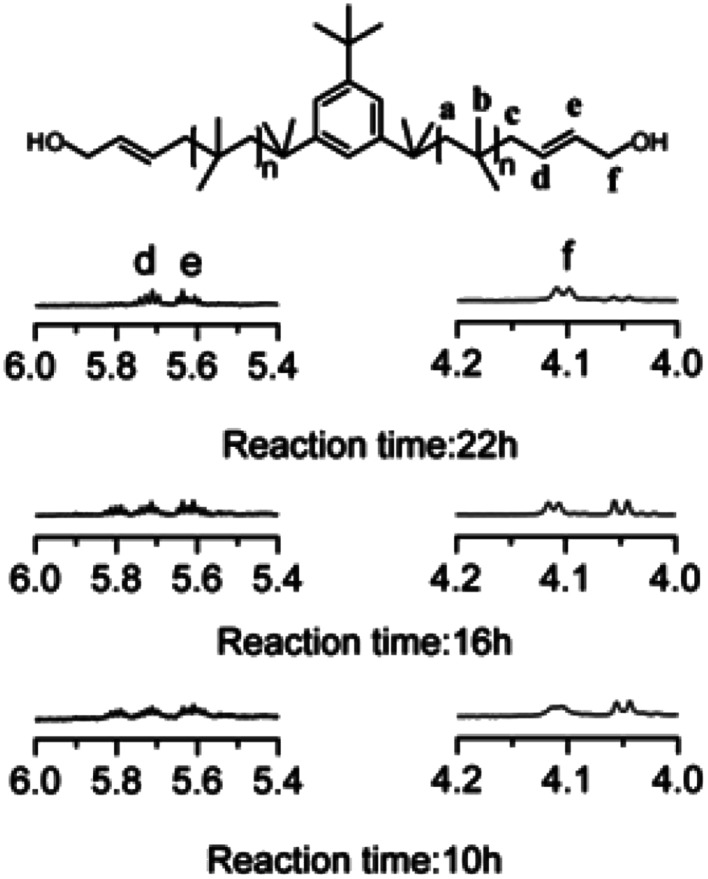
Effect of hydrolysis time on Cl–Allyl–PIB–Allyl–Cl.

**Fig. 11:**
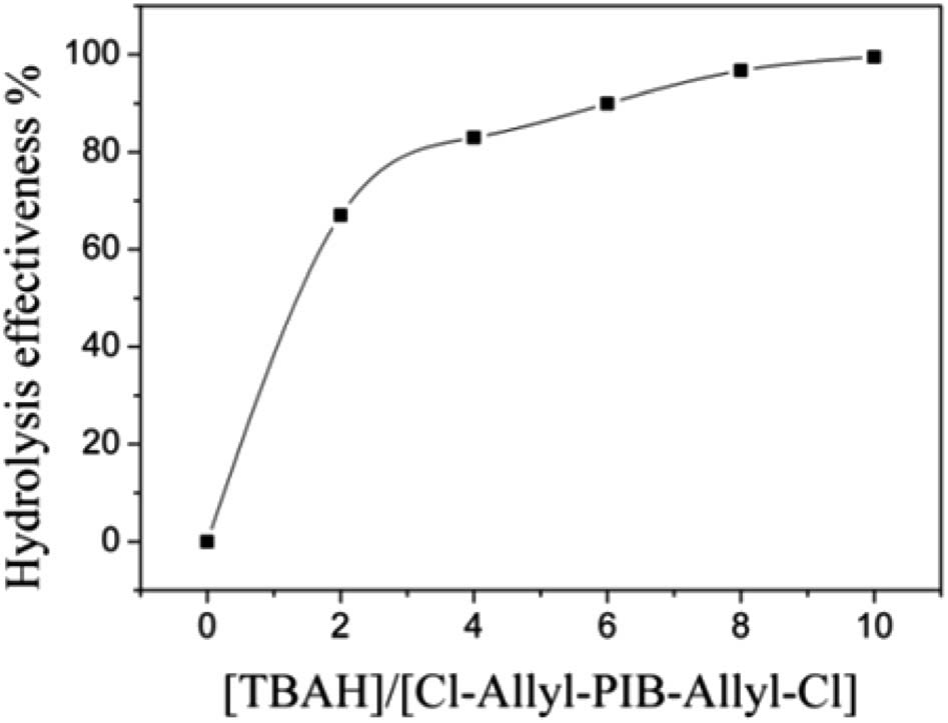
Influence of the ratio of [TBAH]/[Cl–Allyl–PIB–Allyl–Cl] on the effectiveness of hydrolysis during the polymerization of HO–Allyl–PIB–Allyl–OH.

Allyl halides are easily hydrolyzed to the corresponding enols in the presence of an inorganic base. Hence, Cl–Allyl–PIB–Allyl–Cl is a better choice for hydrolytic reactions than Cl–PIB–Cl. The hydrolytic reaction includes homogeneous and heterogeneous reaction systems. The homogeneous reaction system does not require high reaction temperatures, high pressures, or long reaction times. The product of the homogeneous reaction is easier to separate and purify than the product of the heterogeneous reaction system. In the present experiment, a homogeneous system was adopted to produce PIB with high quality hydroxyls at the end groups (HO–Allyl–PIB–Allyl–OH). The hydrolytic reaction of Cl–Allyl–PIB–Allyl–Cl was carried out in a homogeneous THF system to which TBAH was added (Fig. 8) according to the ^1^H NMR spectrum (Fig. 9) of the hydrolysis-based Cl–Allyl–PIB–Allyl–Cl and TBAH. The peaks at 5.81 and 5.61 ppm (–C**H**=C**H**–CH_2_–Cl) increased over time and shifted to a (5.72 ppm) and b (5.63 ppm), which correspond to the H proton in C**H**=C**H**–CH_2_–OH. After 22 h, the chloromethylene proton peak (–CH=CH–C**H**_2_–Cl) at 4.05 ppm disappeared and the hydroxymethylene proton peak (–CH=CH–C**H**_2_–OH) appeared at 4.10 ppm. Thus, according to the ^1^H NMR spectrum, the formation of HO–Allyl–PIB–Allyl–OH could be verified. Fig. 11 shows the hydrolysis effectiveness of the ratio of TBAH to Cl–Allyl–PIB–Allyl–Cl on hydrolysis. Under the premise that the reaction time is set to 22 h (Fig. 10), the hydrolytic effectiveness increases with increasing ratio of reactants. In particular, when the ratio reaches 10, the hydrolytic effectiveness approaches 100%.

### Synthesis of PIB-based polyurethane

**Fig. 12:**
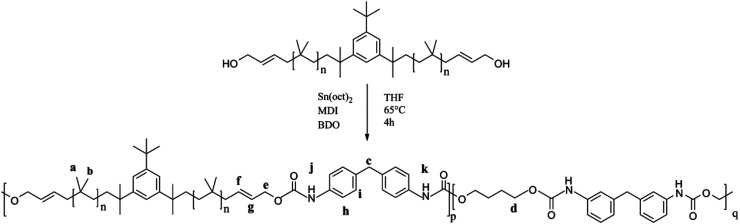
Synthesis of the PIB-based polyurethane.

**Fig. 13:**
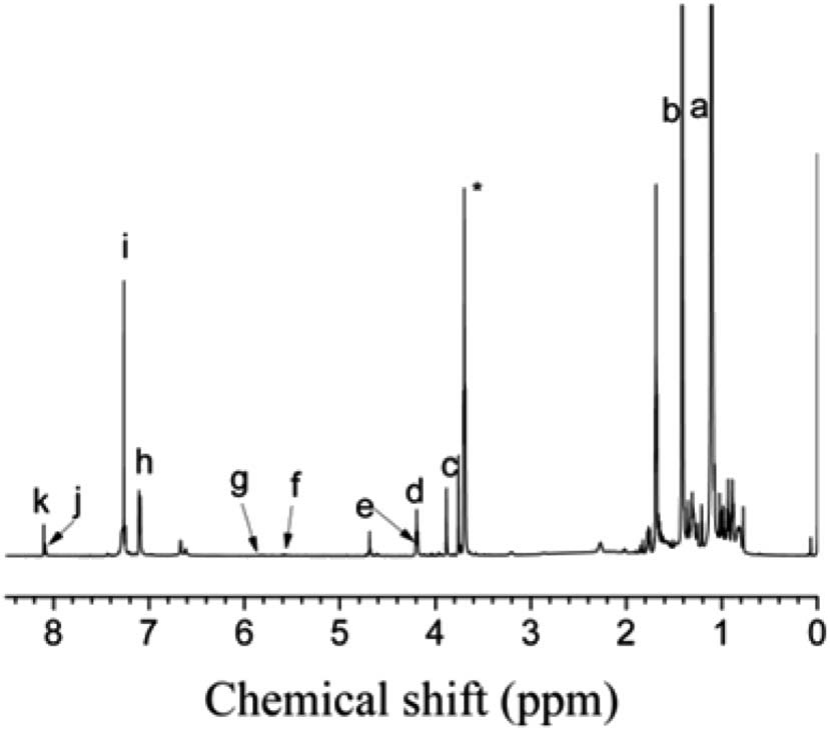
^1^H NMR spectrum of the PIB-based polyurethane. Peaks marked * belong to CH_3_OH.

**Fig. 14:**
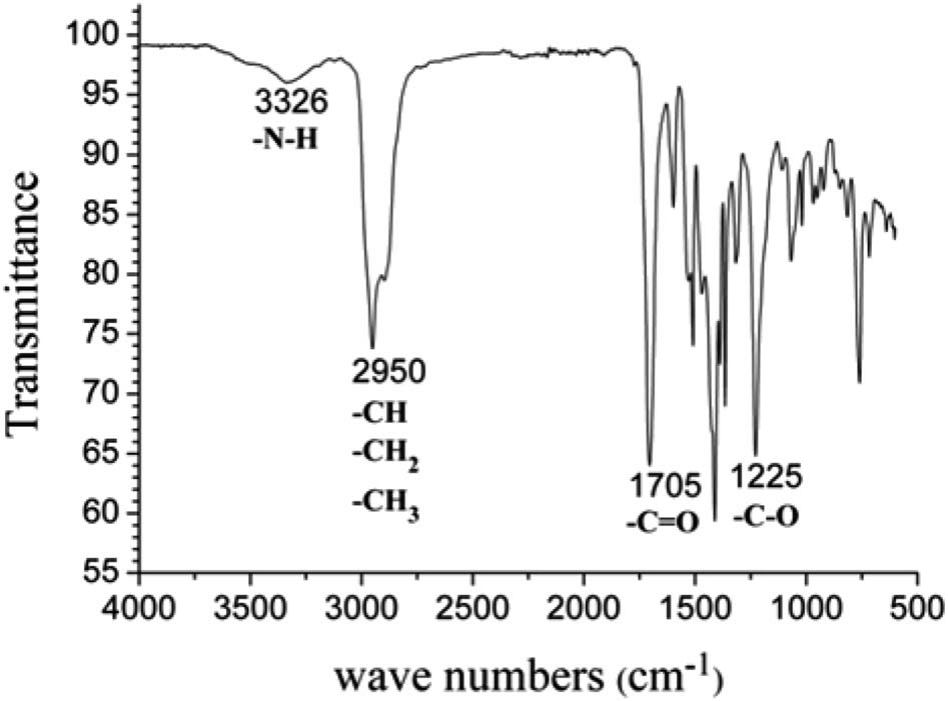
FT-IR spectrum of the PIB-based polyurethane.

As shown in Fig. 12, PIB-based polyurethane is typically produced *via* nucleophilic addition between the hydroxyl group belonging to PIB and the isocyanate group of MDI. BDO, which is added as a chain extender, reacts with MDI to increase the chain length, and PIB replaces the soft segment of polyurethane. Fig. 13 shows the representative ^1^H NMR spectrum of the PIB-based polyurethane. The presence of multiplets (N**H**) at k (8.1 ppm; representing MDI linked to HO–Allyl–PIB–Allyl–OH) and j (8.09 ppm; representing MDI linked to BDO) indicate that BDO acts as the chain extender to bond the hard and soft segments of the PIB-based polyurethane. The multiplets at f (5.63 ppm) and g (5.72 ppm) are due to –CH–C**H**=C**H**–CH– of the allyl. The disappearance of hydroxymethylene protons at 4.10 ppm and the appearance of the multiplet at e (4.20 ppm) due to the methylene protons adjacent to C=C of the allyl indicate the virtually quantitative formation of urethane linkages. The multiplet at d (4.19 ppm) due to the methylene protons adjacent to the oxygen of BDO, the singlet at c (3.82 ppm) due to the methylene protons of MDI between the aromatic rings, and the resonance between i (7.36 ppm) and h (7.03 ppm) reflect the aromatic protons of MDI and the aromatic protons, respectively, of the initiator used for carbocationic polymerization of Cl–Allyl–PIB–Allyl–Cl. The FT-IR spectra of the polymers in Fig. 14 reveal the absence of –NCO absorption at 2225 cm^−1^, which indicates complete conversion. Thus, the ^1^H NMR and FT-IR spectra show that the PIB-based polyurethane elastomer with PIB as the soft segment was successfully synthesized.

**Fig. 15:**
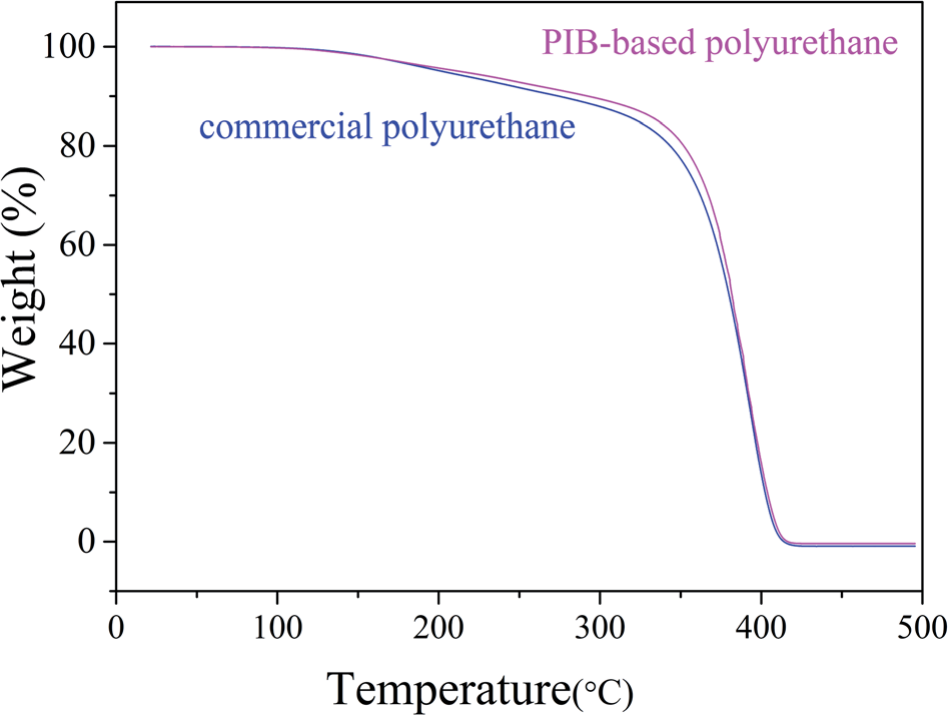
Comparison of the thermal stabilities of PIB-based and commercial polyurethanes.

As shown in Fig. 15, the thermal stability of the synthesized polyurethane was slightly better than that of commercial polyurethane. We investigated the mechanical properties of the PIB-based and commercial polyurethanes using samples processed before the mechanical tests as follows: acidification: immersion in HNO_3_ buffer solution for 10 min at 80 °C; hydrolysis: steam treatment for 96 h at 85 °C; thermal oxidation: air circulation in 128 °C furnace for 48 h. Tensile testing was conducted after washing of the products with deionized water and drying in a vacuum oven for 24 h at 45 °C. Table 1 lists the results of the mechanical test before and after processing. The commercial product had a slightly higher tensile strength than the PIB-based polyurethane due to the presence of flexible PIB segments. However, after processing, the PIB-based polyurethane showed less loss of tensile strength compared with commercial polyurethane. As shown in Table 1, in the acid resistance test, the loss of tensile strength of the PIB-based polyurethane (64.9%) after acidification was less than that of commercial polyurethane (100%), likely because PIB has higher acid resistance than commercial polyurethane. In the hydrolytic stability test, the loss of tensile strength of the PIB-based polyurethane (6.3%) after hydrolysis was less than that of commercial polyurethane (13.6%). Finally, in the thermal oxidative stability test, the loss of tensile strength of the PIB-based polyurethane (7.4%) after processing was less than that of commercial polyurethane (12.1%), according to the FT-IR spectra of polymers, it likely due to the increased adsorption coefficient with increasing strength of H bonding (3326 cm^−1^) in PIB-based polyurethane is higher than in commercial polyurethane, and the defect caused by the polarity of the ester bonds of commercial polyurethane, thereby making it vulnerable for oxidation and hydrolysis.^37–39^ These results indicate that the soft segment of the polyurethane was replaced with PIB and that the PIB-based polyurethane has better acid resistance and hydrolytic and thermal oxidative stability than commercial polyurethane.

**Table 1:** Tensile strength behavior of PIB-based and commercial polyurethanes before and after testing

		Tensile strength (kPa)
**Acid resistance test**		
PIB-based polyurethane	Before test	3824
	After test	1338
	Loss of tensile strength	64.9%
Commercial polyurethane	Before test	4083
	After test	0
	Loss of tensile strength	100%

**Hydrolysis stability test**		
PIB-based polyurethane	Before test	3824
	After test	3583
	Loss of tensile strength	6.3%
Commercial polyurethane	Before test	4083
	After test	3527
	Loss of tensile strength	13.6%

**Thermal oxidation stability test**		
PIB-based polyurethane	Before test	3824
	After test	3541
	Loss of tensile strength	7.4%
Commercial polyurethane	Before test	4083
	After test	3588
	Loss of tensile strength	12.1%

## Conclusions

Cationic polymerization of hydroxyl-terminated telechelic PIB was accomplished in *t*-Bu-*m*-DiCuOMe/TiCl_4_ initiating systems in the presence of D*t*BP *via* living cationic polymerization; here, *t*-Bu-*m*-DiCuOMe was used as the high-efficiency initiator. The living PIB chain was capped by BD to form chlorine-terminated telechelic PIB, which was then hydrolyzed with TBAH to form hydroxyl-terminated PIB. NMR confirmed that the hydrolysis was complete, and nearly 100% conversion of HO–Allyl–PIB–Allyl–OH from Cl–Allyl–PIB–Allyl–Cl was observed. Commercial polyurethane suffers from poor hydrolytic and thermal oxidative resistance. PIB-based polyurethane synthesized by using HO–Allyl–PIB–Allyl–OH as a precursor has greater acid resistance and hydrolytic and thermal oxidative stability than commercial polyurethane.

## Funding

This research was funded by the National Natural Science Foundation of China (No. 51573020), Beijing Natural Science Foundation (No. 2182016,2172022), Scientific Research Project of Beijing Educational Committee (KM201810017008), Project of Petrochina (No. KYWK18002).

## Conflicts of interest

There are no conflicts to declare.

## Supplementary Material
